# The Influence of Family Function on State Anxiety of Chinese College Students During the Epidemic of COVID-19

**DOI:** 10.3389/fpsyg.2021.701945

**Published:** 2021-09-16

**Authors:** Lingping Yang, Miao Wu, Yuqi Wang, Bin Peng

**Affiliations:** College of Public Health and Management, Chongqing Medical University, Chongqing, China

**Keywords:** COVID-19, Chinese college student, anxiety, family function, mediating effect analysis

## Abstract

The Coronavirus Disease 2019 (COVID-19) may affect mental health. There is little research about the influence of family function on the state anxiety of college students in the context of the global pandemic. The study aimed to clarify that generalized anxiety disorder (GAD) and trait anxiety had mediating effects in family function on the state anxiety of Chinese college students following the “stay-at-home” order during the outbreak of COVID-19. This cross-sectional study was conducted online with 1,039 respondents. We analyzed demographics, The State-Trait Anxiety Inventory (ST-AI) and Chinese Family Function Scale (FAD-18), Generalized Anxiety Disorder-7 (GAD-7), and used path analysis to discuss the influence of family function on state and trait anxiety. The results showed that female students’ state and trait anxiety was higher than that of male students (*P* < 0.05). Medical students’ state and trait anxiety was lower than that of literature students (*P* < 0.05). The GAD of the male was lower compared with the female. The score of family function has significant differences in gender, age, education, and region (*P* < 0.05). Gender, family function, state anxiety, trait anxiety, and GAD relate to others (*r* = 0.07∼0.85, *p* < 0.05). The results of fit indices for measurement invariance models showed that the impact of family function among GAD, state and trait anxiety across gender is significantly different (each step *p* < 0.05). GAD and trait anxiety had a complete meditating effect between family function and state anxiety (the proportion of standard indirect mediating effect was 24.94% in females and 36.79% in males). A healthy family function may alleviate GAD and anxiety of college students during the COVID-19 pandemic.

## Introduction

Coronavirus disease 2019 (COVID-19) is an acute respiratory infectious disease caused by the 2019 Coronavirus. As we all know, COVID-19 is such a dreadful disease that has been “raging” on earth from November 2019 up to now (Liu Q. et al., 2020). Thus, many countries have adopted various methods to prevent more people from being infected. The Chinese government has taken many practical measures in the early stage, such as “lockdown” of the cities, and the order to “stay at home” (Wang M. W. et al., 2020). Brooks et al. (2020) reported that quarantine could bring some psychological diseases such as post-traumatic stress symptoms, confusion, and anger. Turkmen et al. (2020) said that lengthy quarantine could increase psychosocial stress and lead to “stress-sensitive” diseases. These studies showed that lengthy-home quarantine might cause ill mental health in the social environment of the epidemic.

The first cause of disability is mental health problems, a significant public health issue worldwide (Ramon-Arbues et al., 2020). In particular, the prevalence of depression and anxiety in developing countries is 10–44%. What is worse, it is the fourth leading cause of morbidity (Azad et al., 2017). Awadalla et al. (2020) noted the marked rates of depression and anxiety in student populations and the obvious potential negative implications for academic study. They can have a negative impact on engagement with their course of study. Students experiencing depression may miss more classes, tests, and assignments, and are more likely to drop courses than their non-depressed peers (Pedrelli et al., 2015). Moreover, it is reported that anxiety has significant adverse effects on academic success, achievement, well-being, and so on (Haller et al., 2015). However, the proportion of depression and anxiety symptoms in college students is very high (Ramon-Arbues et al., 2020). Full-time college students had more time with family members during the “stay-at-home” time. Some evidence showed that family function significantly influences family members’ physiology, psychology, and sociality (Ahlberg et al., 2020). Gonzálvez et al. (2019) showed that good family function would reduce students’ anxiety. However, there are few studies on the impact of such a situation on college students.

Thus, researchers believed that family function might have a significant impact on the generalized anxiety disorder (GAD), state, and trait anxiety of Chinese college students who have to stay at home during the epidemic of COVID-19. Therefore, path analysis was used in the structural equation model (SEM) to discover the relationship of family function, GAD, state anxiety, and trait anxiety.

### Study Aim and Hypotheses

Based on these considerations, the main aim of this study was to investigate the impact of family function on state anxiety by GAD and trait anxiety during the pandemic in Chinese college students. In particular, the purpose of the study was to analyze sociodemographic characterization, the status of family function, state anxiety, trait anxiety and GAD in Chinese college students, the relationship between family function, state anxiety, trait anxiety, and GAD. Moreover, the study explored other sociodemographic variables influence of family function on state anxiety by GAD and trait anxiety. More specifically, the study intended to verify the following hypotheses:

Hypothesis 1 (H1): Females are more anxious than males.
Hypothesis 2 (H2): GAD and trait anxiety mediate family function and state anxiety.
Hypothesis 3 (H3): Gender and major moderated the relationship between family function and state anxiety.

## Materials and Methods

### Setting and Participants

This study is a cross-sectional study to survey the influence of family function on state and trait anxiety of Chinese college students during the COVID-19 epidemic. We have to choose an online survey because of the government’s requirements to stay at home. The questionnaire was distributed *via* Wenjuanxing,^1^ which is an online survey platform; on WeChat (Tencent, Shenzhen, China) and other social platforms (QQ, Tencent, Shenzhen, China). The questionnaire was distributed from March 14 to 21, 2020. The questionnaire link was first sent to the students’ class group and collected by snowball sampling. Then the first respondent forwarded the questionnaire to others.

Specifically, based on the following criteria: age >15, college students, filling time >10 min, respondents without a history of anxiety disorders. The same Internet protocol address, WeChat ID or QQ number were excluded to avoid multiple compilations. The questionnaire with obvious logical errors was eliminated.

### Variables

#### Sociodemographic Characterization

In the sociodemographic survey, respondents were invited to answer simple questions regarding gender, age, education, region, and major. Then they were asked to answer questions related to COVID-19. For example, whom you live with during the epidemic, whether someone around you is infected with COVID-19, the frequency of going out, and the longest time spent at home.

#### Anxiety

State-Trait Anxiety Inventory (ST-AI; Spielberger, 1970) is suitable for evaluating the state and trait anxiety of Chinese College Students. It comprises two blocks (Form Y1 and Form Y2) of 20 items, evaluated in a four-point Likert scale. Form Y1 is used to evaluate transient or temporary anxiety (state anxiety). Form Y2 is used to assesse dispositional or general anxiety (trait anxiety). The score is generated by the sum of the 20 items for each scale. The respondents with a higher score might have a higher anxiety degree. Internal consistency in this study proved to be good (state α = 0.93; trait α = 0.90).

#### GAD

Generalized Anxiety Disorder-7 (GAD-7) is also suitable for evaluating the GAD of Chinese college students. GAD-7 (Spitzer et al., 2006) consists of seven items measuring worry and anxiety symptoms. Each item is scored on a four-point Likert scale (0–3) with total scores ranging from 0 to 21, with higher scores reflecting greater anxiety severity. Scores above ten are considered to be in the clinical range (Spitzer et al., 2006). The GAD-7 has shown good reliability and construct validity (Kroenke et al., 2007; Lowe et al., 2008).

#### Family Function

FAD-18 (Manyan Zhang, 2019) can effectively evaluate the family function of Chinese college students. There are 18 items on the scale, including intimacy, adaptability, control, and growth of four dimensions. Each item is scored on a scale of 0–1 with total scores ranging from 0 to 18, with higher scores reflecting worse family function. Internal consistency in this study proved to be good (total α = 0.87, four dimensions α = 0.82, 0.76, 0.73, and 0.59, respectively).

#### Statistical Analysis

Descriptive and correlation analysis of collected data was analyzed by SPSS23.0 Version (IBM SPSS Statistics, New York, NY, United States). Path analysis in SEM used AMOS 24.0. Quantitative data were analyzed by ANOVA (analysis of variance). Correlation analysis was used to analyze the relationship between factors. The results of the STAI scale were also compared with the healthy norm (Spielberger, 1970) by *Z*-test. Measurement invariance was used to analyze group differences across gender. The mediating effect analysis was used to discover the relationship among family function, GAD, state and trait anxiety. Bootstrapping was used to analyze the mediating effect. Samples were randomly selected 10,000 times. *P* < 0.05 was considered statistically significant.

## Results

### Respondents’ Demographic Characteristics

One thousand two hundred fifty nine questionnaires were submitted. We had eliminated the questionnaires that did not meet the requirements and 1,200 of them were valid. Among the 1,200 questionnaires, 161 questionnaires were excluded due to errors. Finally, there were 1,039 questionnaires left. These respondents were from 30 provinces and province-level municipalities. They were distributed in different majors, grades and living environments. Thus, we are convinced that these respondents can represent Chinese college students.

The results showed that males and females had a significant difference in state anxiety score (*p* < 0.01) and trait anxiety score (*p* < 0.01). It could be easily found that males’ state and trait anxiety scores were lower than females’. What is more, state anxiety and trait anxiety scores were significantly different between medical respondents and other majors (*p* < 0.01). The scores of state and trait anxiety in other majors were higher than those in medicine. Other factors had no difference.

The family function had a significant difference in gender, age, region, and education. That of the male was better than that of the female (*p* < 0.05). What is more, the older students had a better family function (*p* < 0.01). Those respondents with a high degree of education had a better family function (*p* < 0.05). Significantly, the family function was better in the region where the epidemic was not serious (*p* < 0.05). The score of GAD in gender was different. Similarly, the male had lower scores than the female (*p* < 0.05). Detailed results could be found in Table 1.

**TABLE 1:** The demographic characteristics and ANOVA results of the respondents.

**Variable**		**Number**	**Proportion**	***F* value**	**State anxiety**	**Trait anxiety**	**Family function**	**GAD**
	
		**(Total, *n* = 1039)**	**(%)**	***P*-value**	**Mean ± SD**	**Mean ± SD**	**Mean ± SD**	**Mean ± SD**
**Gender**								
	Male	344	33.11		36.84 ± 10.26	38.04 ± 9.86	4.09 ± 2.78	3.87 ± 3.66
	Female	695	66.89		38.36 ± 8.91	39.45 ± 8.90	4.67 ± 3.39	4.38 ± 3.36
				*F* value	6.00	5.37	7.56	5.00
				*P* value	0.01	0.02	0.01	0.03
**Age (years)**								
	≤22	547	52.65		38.09 ± 9.17	39.30 ± 9.09	4.80 ± 3.47	4.23 ± 3.45
	>22	492	47.35		37.60 ± 9.66	38.64 ± 9.41	4.13 ± 2.87	4.18 ± 3.50
				*F* value	0.71	1.34	11.35	0.05
				*P* value	0.40	0.25	< 0.01	0.82
**Region**								
	Severe epidemic city	109	10.49		38.49 ± 10.18	39.97 ± 10.57	4.96 ± 3.28	4.11 ± 3.08
	Moderate epidemic city	664	63.91		37.77 ± 9.28	38.87 ± 9.09	4.57 ± 3.24	4.20 ± 3.51
	Mild epidemic city	266	25.6		37.82 ± 9.42	38.87 ± 9.06	4.02 ± 3.06	4.49 ± 4.05
				*F* value	0.28	0.69	3.98	0.44
				*P* value	0.76	0.50	0.02	0.65
**Major**								
	Medicine	594	57.17		37.48 ± 9.13	38.39 ± 9.06	4.60 ± 3.24	4.11 ± 3.46
	Science and Engineering	296	28.49		37.66 ± 9.75	39.28 ± 9.58	4.24 ± 2.90	4.16 ± 3.38
	Literature	149	14.34		39.74 ± 9.61	40.78 ± 9.10	4.46 ± 3.65	4.70 ± 3.69
				*F* value	5.21	8.27	1.02	2.57
				*P* value	0.02	0.00	0.31	0.11
**Education**								
	Undergraduate	650	62.56		38.14 ± 9.14	39.40 ± 8.96	4.72 ± 3.40	4.20 ± 3.45
	Postgraduate	389	37.44		37.50 ± 9.71	38.48 ± 9.58	4.18 ± 2.94	4.22 ± 3.50
				*F* value	1.21	2.54	7.28	0.01
				*P* value	0.27	0.11	0.01	0.92
**Number of people living together during the epidemic**								
	≤4	767	73.82		37.85 ± 9.52	38.94 ± 9.37	4.53 ± 3.29	4.16 ± 3.52
	>4	272	26.18		37.87 ± 9.09	39.11 ± 8.91	4.33 ± 2.98	4.34 ± 3.32
				*F* value	0.00	0.06	0.74	0.53
				*P* value	0.98	0.80	0.39	0.47
**Live with**								
	Without parents	120	11.55		38.91 ± 9.91	39.79 ± 9.45	4.46 ± 3.45	4.22 ± 3.89
	Single parent	111	10.68		38.41 ± 8.63	39.09 ± 8.35	4.65 ± 3.31	4.31 ± 3.41
	Parents	627	60.35		37.68 ± 9.41	38.89 ± 9.37	4.49 ± 3.23	4.18 ± 3.42
	Parents and grandparents	181	17.42		37.42 ± 9.49	38.73 ± 9.27	4.35 ± 2.94	4.25 ± 3.44
				*F* value	0.83	0.38	0.19	0.05
				*P* value	0.48	0.77	0.89	0.99
**Residential area**								
	Rural	399	38.40		37.99 ± 9.84	39.08 ± 9.44	4.45 ± 3.04	4.20 ± 3.58
	City	640	61.60		37.77 ± 9.13	38.93 ± 9.13	4.49 ± 3.32	4.22 ± 3.40
				*F* value	0.14	0.06	0.04	0.01
				*P* value	0.71	0.81	0.85	0.91
**Frequency of going out**								
	About once a week	373	35.90		38.43 ± 9.56	39.20 ± 9.07	4.57 ± 3.41	4.22 ± 3.37
	About once a half month or more	132	12.70		38.01 ± 8.92	39.74 ± 8.96	5.03 ± 3.72	4.72 ± 3.38
	Never	534	51.40		37.42 ± 9.40	38.65 ± 9.44	4.28 ± 2.91	4.08 ± 3.56
				*F* value	2.54	0.88	2.28	0.51
				*P* value	0.11	0.35	0.13	0.47
**The longest days at home (day)**								
	≤ 10	95	9.14		38.71 ± 7.84	38.85 ± 8.16	4.48 ± 3.03	4.37 ± 3.32
	11–20	294	28.30		37.77 ± 9.02	38.91 ± 9.11	4.37 ± 3.19	4.24 ± 3.30
	≥ 21	650	62.56		37.77 ± 9.78	39.04 ± 9.47	4.52 ± 3.25	4.17 ± 3.57
				*F* value	0.47	0.06	0.19	0.30
				*P* value	0.49	0.80	0.67	0.59
**Is anyone around you infected with COVID-2019?**								
	No	1020	98.17		37.80 ± 9.42	38.96 ± 9.26	4.47 ± 3.22	4.19 ± 3.46
	Yes	19	1.83		40.84 ± 8.06	40.21 ± 8.44	4.79 ± 3.12	5.37 ± 3.79
				*F* value	1.95	0.34	0.18	2.15
				*P* value	0.16	0.56	0.67	0.14

There was no significant difference between the respondents and the healthy norm excluded the score of trait anxiety in females. In this survey, the scores of state and trait anxiety of females were 38.36 ± 8.91 and 39.45 ± 8.90 accordingly, and those of males were 36.84 ± 10.26 and 38.04 ± 9.86. Similar to the healthy norm, the scores of females were higher than those of males in both state and trait anxiety, and the trait anxiety scores of both females and males were higher than state anxiety. Females had a lower score of trait anxiety than the norm (*p* < 0.05). Detailed results could be found in Table 2.

**TABLE 2:** The *Z*-test analysis results of state and trait anxiety compared with norm.

**Anxiety**	**Gender**	**Scores**		***P*-value**
		**Norm**	**Samples**	
State anxiety	Male	36.47 ± 10.02	36.84 ± 10.26	0.49
	Female	38.76 ± 11.95	38.36 ± 8.91	0.38
Trait anxiety	Male	38.30 ± 9.18	38.04 ± 9.86	0.60
	Female	40.40 ± 10.15	39.45 ± 8.90	<0.05

### Multivariate ANOVA

The results showed that interaction between gender and major had no statistical significance (*p* > 0.05). Only gender showed significant difference in state anxiety, trait anxiety, family function, and GAD. Detailed results could be found in Table 3.

**TABLE 3:** The results of Multivariate ANOVA.

**Variable**	**State anxiety**		**Trait anxiety**		**Family function**		**GAD**	
	***F* value**	***P*-value**	***F* value**	***P*-value**	***F* value**	***P*-value**	***F* value**	***P*-value**
Gender	6.03	0.01**	5.40	0.02*	7.56	0.01**	5.02	0.03*
Major	1.63	0.16	2.30	0.06	0.59	0.67	1.89	0.11
Gender*Major	1.44	0.22	1.14	0.33	1.54	0.19	0.97	0.42

*P < 0.05 and **P < 0.01.

### Correlation Analysis

There were positive correlations among gender, family function, state anxiety, trait anxiety and GAD. Correlation coefficient *r* = 0.07 ∼ 0.85 (*p* < 0.05). A detailed description of the results could be found in Table 4.

**TABLE 4:** The results of Pearson correlation analysis among gender, state anxiety, trait anxiety, family function, and GAD.

**Variable**	**Trait anxiety**	**Family function**	**GAD**	**Gender^a^**
State anxiety	0.85***	0.26***	0.65***	0.08*
Trait anxiety		0.32***	0.67***	0.07*
Family function			0.28***	0.09**
GAD				0.07*

*P<0.05, **P<0.01, and ***P<0.001.

*^*a*^Biserial correlation coefficient.*

### Path Analysis

Because it was a saturated model, which means that the number of estimated parameters exactly equal to the elements in the covariance matrix (the degree of freedom is 0), researchers no longer estimated its fit indices; but only focus on its path coefficients. Considering family function as an independent variable, state anxiety as a dependent variable and GAD and trait anxiety as mediating variables across gender, the results of bootstrapping showed that GAD and trait anxiety had complete mediating effects between family function and state anxiety. Gender invariance testing yielded similar models for female and male respondents, with significant chi-square for any step difference (*p* < 0.05). Detailed fit indices for measurement invariance models across gender could be found in Table 5.

**TABLE 5:** Fit indices for measurement invariance models for men and women: baseline (unconstrained), weak (measurement weights), strong (measurement intercept), and strict (measurement residual) (*N* = 1039).

**Parameter**	**Baseline**	**Weak**	**Strong**	**Strict**
Chi^2^ (df)	0.000 (0)	17.639 (6)	35.070 (7)	45.889 (10)
ΔChi^2^ (Δdf)	–	17.639 (6)	17.431 (1)	10.819 (3)
CFI	1.000	0.995**	0.987***	0.983*
RMSEA	–	0.043	0.062	0.059

*P<0.05, **P<0.01, and ***P<0.001.

*CFI, Comparative fit index; RMSEA, Root-mean-square error of approximation.*

**FIGURE 1:**
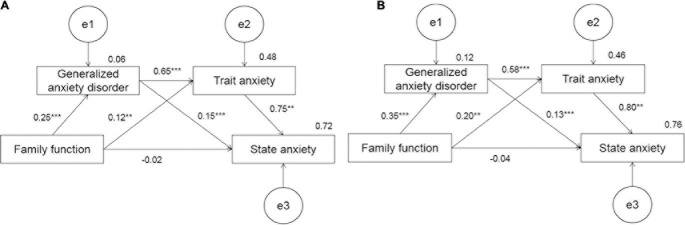
Standard path analysis of family function, GAD, state, and trait anxiety for females and males. **(A)** Female and **(B)** male are included. **P < 0.01 and ***P < 0.001.

The proportion of standard indirect mediating effect was 24.94% in females and 36.79% in males. The estimation of standard direct effect was −0.023 in females (*p* = 0.306) and −0.040 in males (*p* = 0.263). Detailed bootstrapping results of estimated path coefficients could be found in Figure 1 and Table 6.

**TABLE 6:** The results of estimation about standard parameters in path coefficients among family function, GAD, state anxiety, and trait anxiety for females and males.

	**Coefficients (95% CI)**	
**Path**	**Female**	**Male**
GAD← Family function	0.246 (0.171, 0.320)***	0.347 (0.229, 0.450)***
Trait anxiety← GAD	0.654 (0.605, 0.700)***	0.584 (0.506, 0.651)***
Trait anxiety← Family function	0.119 (0.057, 0.179)**	0.196 (0.117, 0.271)***
State anxiety← GAD	0.150 (0.092, 0.207)***	0.135 (0.055, 0.213)**
State anxiety← Family function	−0.023 (−0.066, 0.021)	−0.040 (−0.112, 0.030)
State anxiety← Trait anxiety	0.746 (0.699, 0.7994)***	0.796 (0.716, 0.869)***

**P < 0.01 and ***P < 0.001.

## Discussion

The vast majority of respondents had never been outside. It showed the fact that prophylactic measures implemented by China were effective. Moreover, it was not hard to find that most of the respondents lived with their parents. The study of Gonzálvez et al. (2019) may explain that the respondents with better family function have lower anxiety levels. The results in Table 2 showed no significant difference between respondents, and the norm excluded the trait anxiety of females. The result of Liu X. et al. (2020) was the same as ours. Due to implementing the “stay-at-home” order, college students may be exposed to social media for a longer time, thus receiving more information about COVID-19. Some studies (Li et al., 2020) showed that people might produce vicarious traumatization (VT) in the case of such social events. It is a kind of psychological abnormality indirectly caused by witnessing many cruel and destructive scenes, and the degree of damage exceeds the psychological and emotional tolerance limit of some people (Molnar et al., 2020). However, considering that it has been nearly 4 months since the outbreak of the COVID-19 to this survey, the respondents may have developed compassion fatigue (Hayuni et al., 2019), a psychological self-protection mechanism of the human body. Elena Commodari et al. (Commodari and La Rosa, 2020) reported that Italian adolescents had a low perception of risk of COVID-19, perceived comparative susceptibility and perceived seriousness because of high trust in their good health. What is more, it is reported that good support for prevention and control policies is negatively associated with depression among respondents (Ding et al., 2020). China controlled the epidemic well in a short time. Therefore, that may be one reason why there was little difference between the respondents and the healthy norm.

The score of scales in males was lower than that in females (*p* < 0.05). This may indicate that females may become more anxious and vulnerable to social events, which was similar to the results of Zhong et al. (Bäuerle et al., 2020; Wang C. et al., 2020; Zhong et al., 2020). The state and trait anxiety scores of medical college students are lower than those of literature. Studies showed that (Chang et al., 2020) medical respondents shave excellent professional foundation and a better understanding of COVID-19, while literature respondents suffered more anxiety. A family function is related to gender, age, education, and region. Mainly, the family function performed better in the less severe epidemic region. Therefore, it indicated that family function may be related to COVID-19.

The research of Leslie et al. (2015) and Moe et al. (2021) said that females’ underrepresentation has been linked to gender stereotypes and ability-related beliefs as well as gender differences in specific cognitive abilities. Ceci et al. (2014) reported that females in different majors might be affected by family factors more than males. But our results showed that there is no interaction of gender and major in state, trait anxiety, family function and GAD (*p* > 0.05) and major had no difference in multivariate ANOVA analysis. It may be indicated that gender affected family function on anxiety while major was not.

Although other factors were not significant, we also found some notable points. The results showed that with parents around, less anxiety was experienced. The average scores of state anxiety in respondents who went out frequently were highest. This suggested that those respondents may still be anxious about COVID-19. Our results were similar to those of others (Elrashidi et al., 2018; Ayhan Başer et al., 2020; French et al., 2020; Jacobson et al., 2020; Temsah et al., 2020; Tull et al., 2020).

Generalized anxiety disorder and trait anxiety had complete mediating effects between family function and state anxiety, which indicates that family function has no direct effect on state anxiety in the short term during the epidemic period. It can be significantly influenced by trait anxiety and GAD. However, the research of Shain et al. (2020) said that, in general, state anxiety could be directly influenced by family function. It indicated that COVID-19 might have a more significant impact on family function during “stay-at-home” time. Angelica Moè et al. (Moè and Putwain, 2020) reported that females might be more easily affected by external cause than males. We could quickly find the results from measurement invariance that family function on state anxiety differed across gender. It may be suggested that during the outbreak of COVID-19, the impact of family function on trait anxiety of females was less than that of males, which suggested that females were vulnerable to social events (Kelly, 2020).

### Practical Implications

The theoretical implication of this study is the analysis of how family function affects state anxiety by GAD and trait anxiety across gender. In particular, trait anxiety and GAD had a positive impact on state anxiety. Besides, GAD and trait anxiety are mediating effects between family function and state anxiety. Thus, this justifies the model of this study, which links family function with state anxiety. Moreover, this may offer much evidence for further study.

This study also has social implications. As an essential part of society, a family affected many aspects of family members. Especially, college students dependent on family more than others. Health education can increase the family function among the public. In addition, a healthy family function can alleviate the anxiety and depression of college students.

### Limitations

This study also had limitations. Firstly, the collection of data was conducted by snowball resampling not based on representative sampling. Secondly, there was a significant difference in the number of people in some groups in this study, which may impact the analysis results. Thirdly, some factors had not been observed and not been included in the study, which will affect the final results. Last, among the respondents in our study, few of them had COVID-19 cases around them. Therefore, the study’s conclusion may only apply to Chinese college students who do not have COVID-19 cases around them.

## Conclusion

Some significant factors related to this study were also found. Among Chinese college students, females were more likely to be affected by social events, and aggravating their degree of anxiety. Furthermore, we also found that medical college students were less anxious about pandemic infectious diseases than literature students. Those who had a better family function had less anxiety. The family function had a significant impact on trait anxiety and GAD, and indirectly impacted upon state anxiety. Thus, the mental health of females should be paid more attention to. A better family function can alleviate the anxiety of college students during the epidemic of COVID-19. Generally, the significance of this study is profound.

## Data Availability Statement

The raw data supporting the conclusion of this article will be made available by the authors, without undue reservation.

## Ethics Statement

The studies involving human participants were reviewed and approved by the Ethics Committee of Chongqing Medical University. Written informed consent from the participants’ legal guardian/next of kin was not required to participate in this study in accordance with the national legislation and the institutional requirements.

## Author Contributions

BP and LY conceived the presented idea and performed the data analysis. LY, MW, and YW performed material preparation and assisted with the recruitment process and distribution of the surveys. LY performed data management. All authors contributed to the study’s conception and design, writing and review of the manuscript, and read and approved the final manuscript.

## Conflict of Interest

The authors declare that the research was conducted in the absence of any commercial or financial relationships that could be construed as a potential conflict of interest.

## Publisher’s Note

All claims expressed in this article are solely those of the authors and do not necessarily represent those of their affiliated organizations, or those of the publisher, the editors and the reviewers. Any product that may be evaluated in this article, or claim that may be made by its manufacturer, is not guaranteed or endorsed by the publisher.

## Footnotes

1
www.wjx.cn
